# Fractional chaotic maps based short signature scheme under human-centered IoT environments

**DOI:** 10.1016/j.jare.2020.08.015

**Published:** 2020-09-09

**Authors:** Chandrashekhar Meshram, Rabha W. Ibrahim, Ahmed J. Obaid, Sarita Gajbhiye Meshram, Akshaykumar Meshram, Alaa Mohamed Abd El-Latif

**Affiliations:** a Department of Post Graduate Studies and Research in Mathematics, Jayawanti Haksar Government Post Graduation College, College of Barkatullah Vishwavidyalaya, Betul, M.P. 460001, India; b Informetrics Research Group, Ton Duc Thang University, Ho Chi Minh City, Viet Nam; c Faculty of Mathematics & Statistics, Ton Duc Thang University, Ho Chi Minh City, Viet Nam; d Department of Computer Science, Faculty of Computer Science and Mathematics, University of Kufa, Iraq; e Department for Management of Science and Technology Development, Ton Duc Thang University, Ho Chi Minh City, Viet Nam; f Faculty of Environment and Labour Safety, Ton Duc Thang University, Ho Chi Minh City, Viet Nam; g Department of Applied Mathematics, Yeshwantrao Chavan College of Engineering, Nagpur, M.S., India; h Department of Mathematics, Faculty of Education, Ain Shams University, Roxy, Cairo, Egypt; i Department of Mathematics, Faculty of Arts and Science, Northern Border University, Rafha, Saudi Arabia

**Keywords:** Fractional chaotic map, Chaotic systems, Short signature scheme, Internet of Things (IoT), Confidentiality, Probability security analysis

## Abstract

**Introduction:**

The Internet of Things (IoT) comprises of various smart devices for the sharing of sensed data through online services. People will be directly contacted to check their health parameters and the reports will be collected centrally through smart devices. The requirement is protection of messages during the exchange of data between sender and receiver in order to tackle human malicious attacks. Various signature-based schemes are discussed in the literature to provide secure communication. Smart devices however require lightweight tasks by ensuring critical safety strengths. An important problem in the signature based method is that it incurs more computational expenses for signing and verification process in large numbers.

**Objectives:**

In this study, we introduced an efficient Short Signature Scheme (SSS) that uses Fractional Chaotic Map (FCM) for secure communication in IoT based smart devices, the security of which is closely related to a random oracle based on FCM assumption.

**Methods:**

In this study, we have designed new short signature scheme using FCM. The presented scheme consist of four sub-algorithm as follows: setup, key generation, signing and verification. We have used less rigorous operations based on the FCM to carry out signing and verification procedures, similar to human signing on valid documents and then verifying them as per witness.

**Results:**

The proposed SSS offers a better security assurance than currently established signature schemes. The key advantage of the SSS over the DSA schemes is that at the verification stage and signing period it takes less computation; it retains the degree of protection. The presented SSS takes less bandwidth for storage, communication, and computing resources; particularly applicable to wireless devices and smart cards.

**Conclusion:**

We concluded that the uses of fractional chaotic maps is more effective for secure communication in human-centered IoT to present a provably secure short signature technique.

## Graphical abstract

**Figure:**
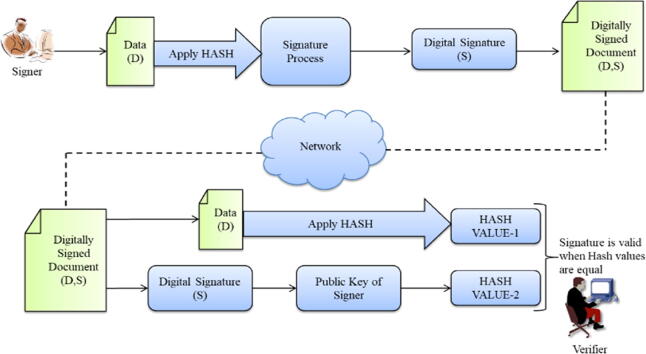

## Introduction

In this era of the Internet of Things (IoTs), in which various device types are connected to the Internet. Such devices can be household appliances, agricultural equipments, manufacturing, energy meter, industrial machinery, healthcare monitoring machinery, mining sensors, surveillance system, environmental equipment, smart grids and smart city, etc. which includes Machine-to–Machine model. With the advent of IoT enable devices, monitoring or control of various types of systems on the tips of the fingers has become very easy. IoT devices are smart enough to share and exchange data for cloud storage over a public internet. IoT is an effective method for applying to domain varieties and proves the vital function by providing substantial advantages.

**Fig. 1:**
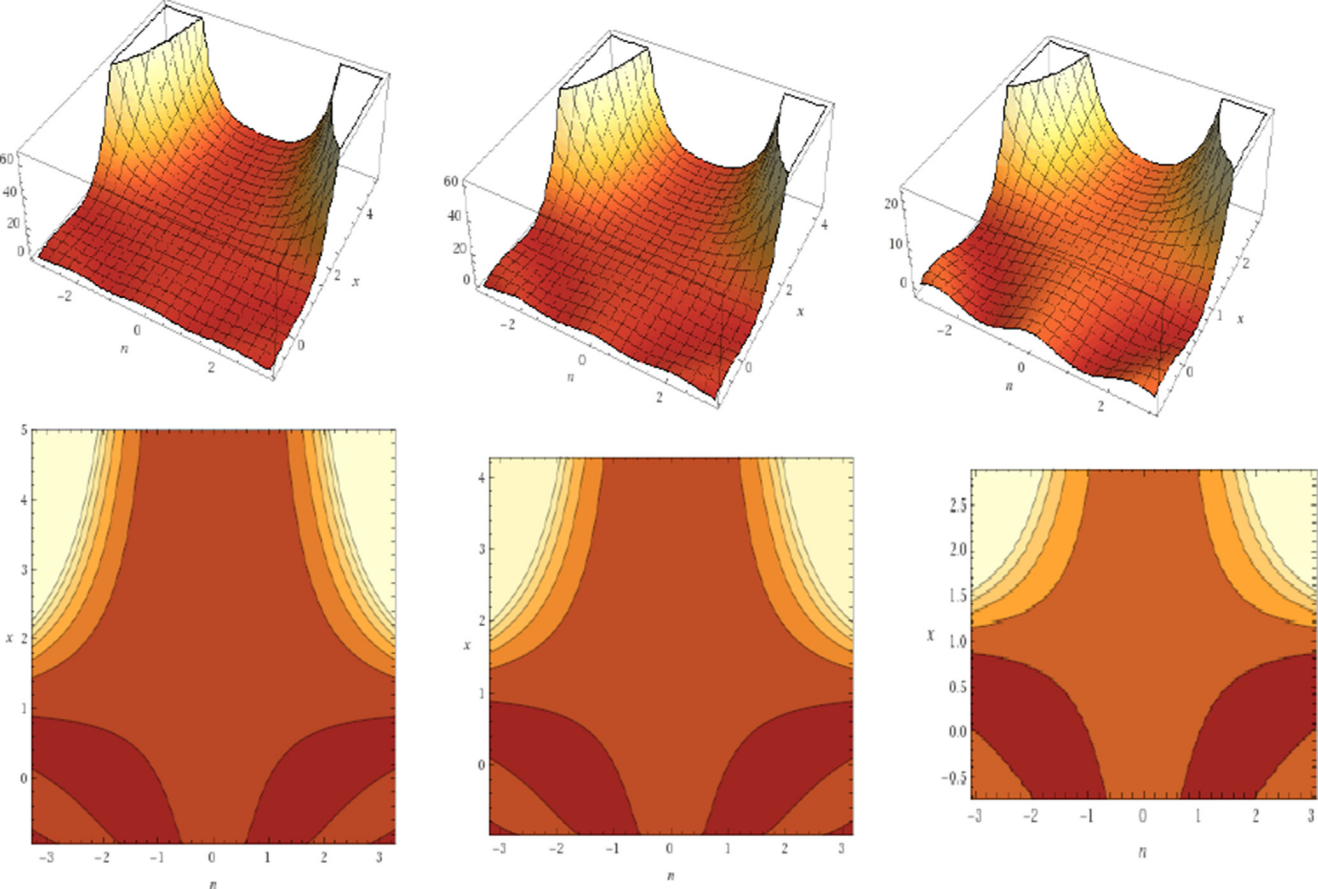
3D-fractal Chebyshev polynomials for α = 0, 0.5 and 0.75 respectively.

Some acknowledged literature are [1], [2], [3], [4], [5], [6], [7], [8], the application of IoT witness in various domains ranging from manufacturing automation to healthcare. Moreover, every attempt is made to improve hardware interfaces, software, improved communication, and less focus is on user interaction and experience, and protection and privacy policies. This means, less significance is given to human related Internet of Things. Subsequently, we investigate human-centered IoT enabled device to offer more preference to human viewpoint in technology. Human-centered IoT is an upcoming filed of research connects to various aspects of life includes smart cards, e-commerce, business, healthcare, and sensitive private data. That means, the human-related Internet of Things is given less significance. Subsequently, we have investigated devices enabled by human-centered IoT to offer more preference in technology to the human viewpoint. Human-centered IoT is an upcoming field of research connected to various aspects of life that includes smart cards, e-commerce, business, healthcare, and sensitive private data. Nonetheless, the design of human-centered IoT [9] offers many opportunities and challenges. Thus, it not only is focuses on IoT system performance, integration, communication and interoperability, but more emphasis is placed on user application features, user need, and human-centered IoT motivation (see Fig. 1).

Smart factories have emerged as a result of the Industry 4.0 revolution and are capable of intelligently managing data produced from the overall production system [10]. Tracking or labeling of items or objects in this scenario is often achieved by RFID tags, QR codes, and barcodes named as labels or tags. The challenges are the incorporated of too many practices in an IoT-centric human world. So we have to address all these challenges to build productive 4.0 industry with human centric applications using smart labels. We are witnessing that human participation in human-centered IoT-based applications in which the design approach adopted focuses less on devices and more on the human-centered [11], [12]. In human-centered IoT, information is exchanged over the public communication channel through various devices. Thus, fraudulent practices occur to steal or change the information. Because of this, the major challenge is preserving confidentiality and privacy during transmission time. Hence, for information exchange, we need more effective and reliable security mechanism. IoT systems are resource-constrained and heavy computing resources lose out. Radwan et al. [13] presented the concept of the synchronization with active control technique of different fractional order chaotic systems. Based on the switching parameters, four different cases of synchronization are also introduced. Ibrahim et al. [14] discussed some symmetric conformable fractional derivatives of complex variables for fractional chaotic maps generalizations. The standard DSA such as RSA [15], ElGamal [16], ECDSA [17], and bilinear pairing [18] are therefore not necessary to apply. We need a quick and lightweight short-size signature security scheme for IoT. It does, however, take time to check the bilinear maps used by the short-signature schemes based on pairing. Moreover, such short signature schemes are not as computationally efficient as the signature schemes of the DSA-type. Hence the storage capacity of pairing-based signatures comes at the expense of losing computational performance. Vaidyanathan et al. [19] introduced a novel 3D jerk chaotic system with one-quadratic nonlinearity and two-cubic nonlinearities designed to produce complex chaotic signals, and addressed voice encryption applications. Vaidyanathan et al. [20] proposed a new model of hyperchaotic temperature fluctuations and described its modeling, and also discussed the characteristics of the new model of hyperchaotic temperature fluctuations, such as its phase portraits, rest points, symmetry, invariance, characteristic exponents of Lyapunov, bifurcation analysis etc. Explain it for image encryption application just briefly. Mobayen et al. [21] introduced 3-D chaotic system with a closed equilibrium point curve, which has the form of a boomerang and modeled the theoretical system's electronic circuit implementation to test its feasibility. Also addressed the sound encryption applications.

A short signature scheme using a chaotic map is more efficient and costs less in terms of computation. Consequently, we have adopted chaotic maps for human-centered IoT’s proposal for a short signature security scheme. Chaotic maps are used in [22] to introduce the authentication scheme for ID-based digital signature. Schemes security promise based on the assumptions of chaotic maps hardness (Diffie-Hellman) and difficulties (DL). In 2016, Gao et al. [23] presented an authentication scheme based on chaotic maps for wireless body area network in which health data was recorded and monitored. The cost of multiplication and exponential computation was achieved with reduced communication cost. User confidentiality was the key element in sharing of information during authentication. An anonymity which preserves authentication scheme is shown in [24]. AVISPA was used for analyzing and verifying security. Compared to other approaches the enhanced performance was recorded. Meshram et al. [25], [26], [27] proposed more efficient authentication schemes using extended chaotic maps. The results obtained in these schemes are testimony to the suitability of chaotic maps as the good choice for proposing a new security scheme.

### Motivation

While some researchers have proposed security mechanisms, they are not lightweight enough to meet the IoT based system’s needs. In this paper, we have proposed an efficient secure and lightweight short signature scheme using fractional chaotic maps which provides security under adaptive chosen-message attack (CMA) in random oracle model.

Recently, Mughal et al. [43] presented a digital signature scheme using complex numbers for providing secure communication among smart devices in human-centered IoT based systems but, have not discussed its security in any standard security model as discussed in the previous literature. A variety of signature-based schemes are discussed in literature to provide secure communication, but smart devices need more lightweight operations by ensuring the required security strengths. Due to the large real numbers required for signature and verification processes, the main problem during signature-based approaches is the computational overhead. This paper presents a lightweight short signature scheme using fractional chaotic maps for providing secure communication between smart devices in human centered IoT. We have used less extensive operations to achieve processes of signing and verification, as human beings do signatures on legal documents and then verify as per witness. The presented scheme is secure under adaptive chosen-message attack (CMA) in random oracle model.

### Contribution

This paper presents an efficient provably secure short signature scheme using fractional chaotic map for smart devices in human centric IoT. During verification and signing operations, it uses the less detailed operations based on fractional chaotic maps to generate security credentials. The main advantage of this strategy over the DSA signature scheme is a one-fourth reduction in the verification process as well as signature frequency. The methodology is illustrated with simple step-by-step, outstanding principles to prove proof of notion. In DSA-based systems, this eliminates overhead computation and communication, and coordination along with improved flexibility compared to existing detailed operations based on real number. However, we show the reliability of the proposed SSS is closely linked, if not strongly, to the difficulty of solving fractional chaotic maps. Under adaptive chosen attacks in ROM, an efficient security proof exists for unforgeability, i.e. the presented scheme provides superior security guarantees than the existing other signature schemes. The scheme presented does not use pairings resulting in effortless implementation and higher performance, nor is it relying on the relatively untested and recent assumptions of hardness associated with pairing-based cryptography. Results show that our methodology presented is less time consuming than equivalents for the verification and signature process. It requires less time to check the variations in the length of the message, less communication costs needed for signature messages, fewer bytes exposed by undermining devices and less ability to compromise midway devices.

Road map of article: Section ‘Related materials’ describes the definition and terminology associated with the presented scheme. The proposed new scheme based on fractal calculus to generalize the Chebyshev polynomial are listed in Section ‘PROPOSED SHORT SIGNATURE SCHME (SSS)’. Section ‘Security analysis and discussion’ explains the security target of signature schemes, security models and provably security in ROM, and we are also presenting a reductionist proof of security against forgery that occurs under the adaptive chosen message attacks (EUF-CMA) in ROM. Section ‘Performance comparison’ describes the study by which other similar recent schemes are contrasted with the scheme proposed. Finally, Section ‘Conclusion’ stretches the conclusions.

## Related materials

In this segment, we have highlighted Chebyshev polynomial and fractional chaotic maps subsequently we would use in the proposed technique. We will then define some necessary notations used in the article (see Table 1).

**Table 1:** . List of notations.

Æ¨	Private Key
	Public Key
$T^{\alpha }$	Fractal Chebyshev chaotic maps
r	Random number per message
,	One Way Hash Functions
Ïº	Message
*D*	1st parameter of signature
	2nd parameter of signature
	Digital Signature
q	Large prime number of bit length
p	Large prime factors of q-1

### Chebyshev chaotic transforms

We reviewed Chebyshev sequential polynomials (CSP) (see [28]) and assessed their operatory. CSP $T_{r}\left(\tau \right)$ is a polynomial of n-degree in the variantτ. Let τ∈[-1,1] be the version, and let n be an integer. In general, CSP stated as follows:

$$T_{n}\left(\tau \right)=cos\left(n\times \cos^{-1}\left(\tau \right)\right),$$

$$T_{0}\left(\tau \right)=1$$

$$T_{1}\left(\tau \right)=\tau$$

$$T_{n}\left(\tau \right)=2\tau T_{n-1}\left(\tau \right)-T_{n-2}\left(\tau \right);n\geq 2$$

In this case, the functional $\cos^{-1}\left(\tau \right)$ and cos(τ) represented as $\cos^{-1}:\left[-1,1\right]\to \left[0,\pi \right]$and$cos:R\to \left[-1,1\right]$.

There are two main properties of CSP [25], [26], [29], [30], [31], [32]: chaotic properties and semi-group properties.

(1) The chaotic possessions: The CSP transform demarcated as $T_{r}:\left[-1,1\right]\to \left[-1,1\right]$ with degreen>1, is a chaotic transform connected to the functional (invariant density) $f^{*}\left(\tau \right)=\frac{1}{\left(\pi \sqrt{1-\tau^{2}}\right)}.$
(2) The possessions of what is calling semi-group satisfies the following equalities:

$T_{w}\left(T_{l}\left(\tau \right)\right)=cos\left(w{cos}^{-1}\left(cos\left(l{cos}^{-1}\left(\tau \right)\right)\right)\right)=cos\left(wl{cos}^{-1}\left(\tau \right)\right)=T_{lw}\left(\tau \right)=T_{l}\left(T_{w}\left(\tau \right)\right)$, where w and l are positive integers and τ∈[-1,1].

Chebyshev polynomials have two tests that in polynomial time considered handling:

(1) The discrete log's (DL) assignment is to find the integer w with the end goal $T_{w}\left(\tau \right)=y$ given two components τand y.
(2) Because of three componentsτ, $T_{w}\left(\tau \right)$, and $T_{l}\left(\tau \right)$, the Diffie-Hellman problem (DHP) assignment is to measure the $T_{wl}\left(\tau \right)$ element.

### Fractional Chebyshev polynomials (FCP)

Fractional discrete systems have a most important benefit over their conservative complements due to the infinite memorial feature, which agrees for more flexibility in demonstrating and indicates a higher degree of chaotic performance. We have confidence in the fractional calculus approaches and fractional discrete formulation that will give us a recovering explanation of discrete fractional maps. From our research, we discovered that the fictionalized standard map could also be employed in the information security field. In this section, we aim to formulate the *Fractional Chebyshev Polynomials.*

Assume the fractional (arbitrary) number α∊[0,1]. An operator $\delta^{\alpha }$ is fractal derivative if and only if [30]

$$\delta^{\alpha }\vartheta \left(x\right)=\lim_{x\to x_{0}}\frac{\Delta^{\alpha }\left(\vartheta \left(x\right)-\vartheta \left(x_{0}\right)\right)}{{\left(x-x_{0}\right)}^{\alpha }}=\Gamma \left(\alpha +1\right)\left(\vartheta \left(x\right)-\vartheta \left(x_{0}\right)\right).$$

The fractal integral corresponds to $\delta^{\alpha }$ is defined by

$$I^{\alpha }\vartheta \left(x\right)=\frac{1}{\Gamma \left(\alpha +1\right)}\int_{a}^{b}\vartheta \left(x\right){\left(dx\right)}^{\alpha }.$$

Note that

(1) $$I^{\alpha }\vartheta \left(x\right)=\frac{{\left(b-a\right)}^{\alpha }}{\Gamma \left(\alpha +1\right)}\vartheta \left(x\right),\;a\leq x\leq b.$$

By employing the concept of Fractal Calculus to simplify the polynomial$T_{n}\left(\tau \right)$, we can attain the subsequent structure:

(2) $$I^{\alpha }T_{n}\left(\tau \right):=T_{n}^{\alpha }\left(\tau \right)=\frac{{\left(2\right)}^{\alpha }}{\Gamma \left(\alpha +1\right)}T_{n}\left(\tau \right),$$

Eq. (2) is named the Fractal Chebyshev polynomials (FCP). Formula that is more frequent can be seen in the following result:

Proposition 2.1 *The FCP fulfills the frequent associations*

(3) $$T_{n}^{\alpha }\left(\tau \right)=\frac{{\left(2\right)}^{\alpha }}{\Gamma \left(\alpha +1\right)}\left(2\tau T_{n-1}\left(\tau \right)-T_{n-2}\left(\tau \right)\right).$$

Proof. Connection (2) with the frequent formula implies that

$$T_{n}\left(\tau \right)=2\tau T_{n-1}\left(\tau \right)-T_{n-2}\left(\tau \right);n\geq 2$$

we have

$$T_{n}^{\alpha }\left(\tau \right)=\frac{{\left(2\right)}^{\alpha }}{\Gamma \left(\alpha +1\right)}T_{n}\left(\tau \right)$$

=$\frac{{\left(2\right)}^{\alpha }}{\Gamma \left(\alpha +1\right)}\left(2\tau T_{n-1}\left(\tau \right)-T_{n-2}\left(\tau \right)\right).$

Note that when α→0, we have the main ordinary result, which can be seen in [33].

Proposition 2.2 *The semi-group possessions clamps for FCP positioned on interval (-∞,∞).*

Proof. Let $h=\frac{{\left(2\right)}^{\alpha }}{\Gamma \left(\alpha +1\right)}.$ By Proposition 2.1, we obtain

$$T_{n+2}^{\alpha }\left(\tau \right)=\frac{{\left(2\right)}^{\alpha }}{\Gamma \left(\alpha +1\right)}\left(2\tau T_{n+1}\left(\tau \right)-T_{n}\left(\tau \right)\right).$$

The above preparation proposes an adjustment equation (disconnected equation) which has a typical principle

$$\omega^{2}-2h\omega +\mu_{1}=0$$

Satisfying the relations

$$\omega_{1}+\omega_{2}=2h,\;\omega_{1}\omega_{2}=\mu_{1},\;\omega_{1,2}=h\pm \sqrt{h{-\mu }_{1}}.$$

A computation yields that

$$T_{n}^{\alpha }\left(\tau \right)={{(\omega }_{1}}^{n}+{\omega_{2}}^{n})/2$$

$$=\frac{{\left(h+\sqrt{h^{2}{-\mu }_{1}}\right)}^{n}+{\left(h-\sqrt{h^{2}{-\mu }_{1}}\right)}^{n}}{2}$$

=∑m=0[n/2]nmhn-2m(h2-μ1)m

A computation yields that

$$T_{k}^{\alpha }\left(T_{n}^{\alpha }\left(\tau \right)\right)={{(\tau }_{1}}^{k}+{\tau_{2}}^{k})/2$$

$$\tau_{1}+\tau_{2}=2T_{n}^{\alpha }\left(\tau \right),\;\omega_{1}\omega_{2}=\mu_{1}.$$

Hence, we have the important relation

$$T_{k}^{\alpha }\left(T_{n}^{\alpha }\left(\tau \right)\right)=T_{n}^{\alpha }\left(T_{k}^{\alpha }\left(\tau \right)\right)=T_{\mathrm{kn}}^{\alpha }\left(\tau \right).$$

The closed form expression for Chebyshev polynomials of any order is

(4) Tix=∑j=0[i/2](-1)ji2jxi-2j(1-x2)j

where [i/2] is the integer part of (i/2). Then the FCP becomes

$${T_{i}}^{\alpha }\left(x\right)=\frac{{\left(2\right)}^{\alpha }}{\Gamma \left(\alpha +1\right)}\left(2xT_{i-1}\left(x\right)-T_{i-2}\left(x\right)\right)$$

=2αΓα+12x∑j=0i/2-1ji-12jxi-2j-11-x2j-∑j=0i/2-1ji-22jxi-2j-21-x2j

## Proposed short signature schme (SSS)

**Fig. 2:**
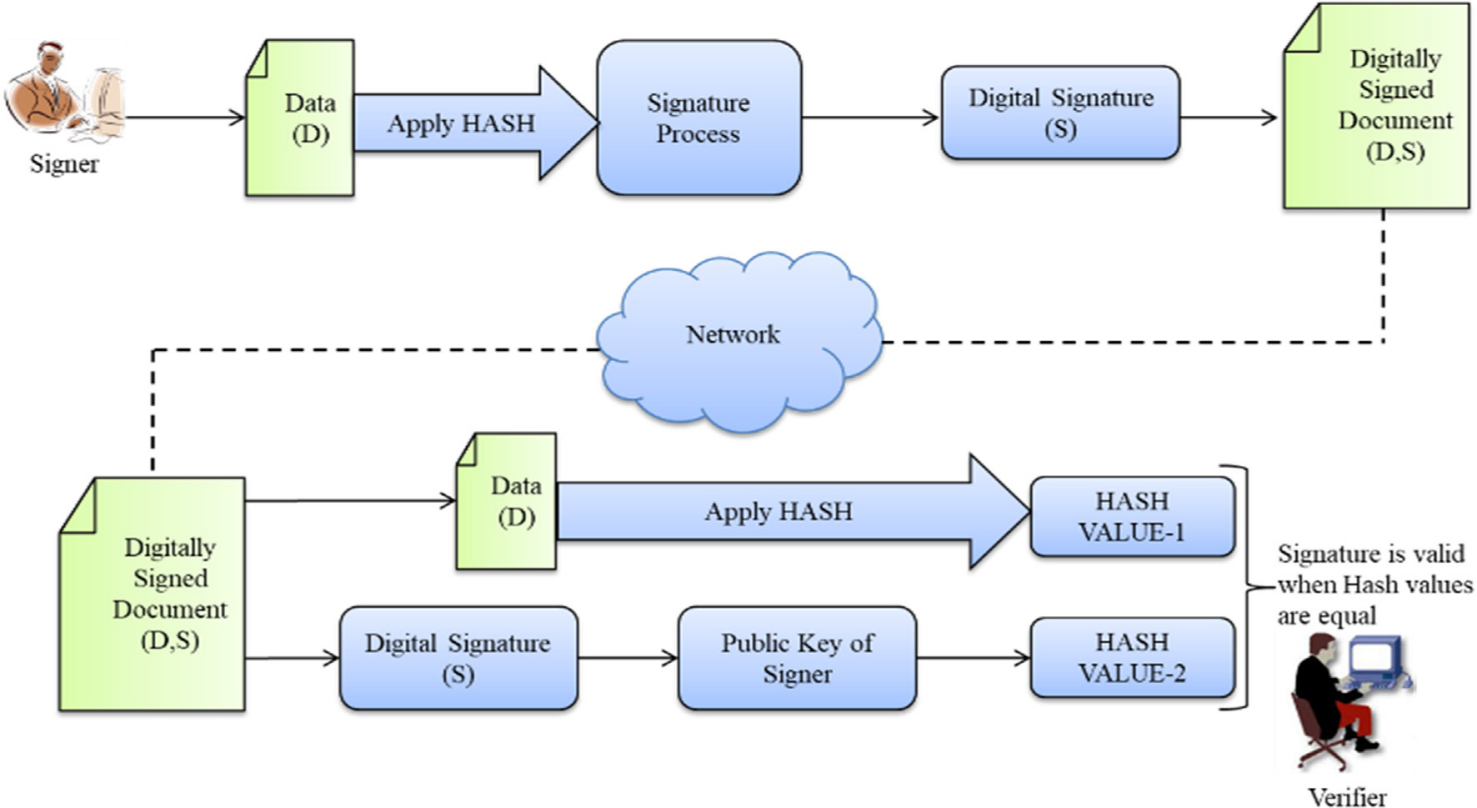
Digital Signature and Verification Process.

In this section, we presented a secure FCM based SSS under the environment of IoT. The presented scheme as follows (see Fig. 2):

• *Setup*: Let q and p be huge prime numbers with p|(q-1). Similarly let $G_{É¡,q}=\left\{{É¡}^{0},{É¡}^{1},...{É¡}^{p-1}\right\}$ be a subgroup with prime order q of the multiplicative group $Z_{q}^{*}$, where É¡ is a generator with prime order p. Let  and  be one way hash functions where

**Figure:**
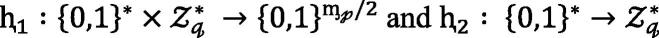

We will omit the “(modq)” and “(modp)” markers for notational convenience. We denote 's bit length by  and . The notation $b\overset{R}{\leftarrow }S$ implies that an is selected randomly from a set S at random. We are now explaining the SSS in full detail.

• Key generation algorithm
1. Picks an arbitrary $Æ¨\overset{R}{\leftarrow }Z_{p}^{*}$ as the private key.
2. The corresponding public key is .
• *Signing algorithm*: The inputs are the private key Æ¨, the public key  and a message $Ïº∊{\left\{0,1\right\}}^{*}$.
1. First choose an arbitrary $r\overset{R}{\leftarrow }Z_{p}^{*}$.
2. Computes  and .
3. The signature of the message m is .
• *Verification algorithm*: The inputs are the message Ïº, the public key  and the signature .
1. Computes  and
2. If $D=D^{\text{'}},$ verification outputs valid, otherwise it outputs in valid.

*Consistency of the proposed scheme*: Because  and  imply  and  so . Hence, the signature  produced by the signing algorithm is always valid.

## Security analysis and discussion

At the very outset, the prototype of the security inspection for presented short signature schemes is observed. Secondly, the concept of the random oracle and the “provable security” are investigated. Finally a near-reductionist method is brought forth to prove that SSS is safe to prevent existential forgery in the setup of chosen attacks in ROM assuming FCM are difficult under human-centered Internet of Things environments.

### Security frameworks and provably security analysis

The primary safety principles of the short signature schemes, the first of its kind technique to use, were described by Goldwasser et al. [35]. Universal forgery, existential forgery (EUF), and absolute break are the three types of attacks chosen by the enemy. However, to destabilize the signature's security the strategies used by enemy may be different. Attacker has the basic knowledge of the signer’s public key in the First instance. Secondly attacker has access to a set of accurate pairs such as message and signature. On the basis of earlier obtained feedback of the questions, the adaptive chosen-message attack (CMA) authorizes the attacker to use the signer’s sign for some of his/her chosen message. In order to avoid many other formerly signatures to correspond to single given message the algorithm for signature generation has to be definite in nature. The attacker is allowed to ask for each message at most one signature due to compromised defensive system in Single-occurrence adaptive chosen-message attack (SO-CMA). The implications of [36], [37] are essential to be pursued for a “ROM” to confer authenticated verified security for the cryptosystems. Hash functions specifically arbitrary items, the hash function is exploited as an oracle to generate a random number for the fresh inquiry. An attacker utilizes a reductionist method of a logical assumption-contradicting method. Probabilities are judged on both guesses and random oracles. A well-constructed hash function generally doesn't bring out random responses practically. As a result, the value of the proofs performed in ROM is contentious. Reformed “artificial” equivalent which are “probably secure” in ROM are described in [38]. However, if the short signature scheme (SSS) needs to be protected a random-model security proof is required to be followed.

Definition 4.1 (SSS’s Existential CMA Security): We deduce that a short signature scheme when running for t steps is shattered by , probabilistic algorithm and gives way to the creation of adaptive inquiries of  to the hash function oracles. The place where probability is based on coins of *A*, Gen algorithm, Sig algorithm, and hash function oracles, *A* for some message M with  probability creates fake signature, demanding signatures () for adaptively selected messages, ().

When  is hard to be ruined by any counterfeiter, the short signature scheme is ()-secure.

Definition 4.2 (FCM Assumption):, If  runs in a maximum of t steps and computes the fractional chaotic maps ${\mathrm{FCM}}_{É¡,q}\left(T_{b}^{\alpha }\left(É¡\right)\right)=b$ given input (É¡,q,p) and $T_{b}^{\alpha }\left(É¡\right)$ with ε probability where probability is based on uniformly selected coins of  and b from $Z_{q}^{*}$, , probabilistic algorithm is said to ()-split FCM in a group$G_{É¡,q}$

Here it can be said that group $G_{É¡,q}$ is a ()-FCM group unless algorithm in group $G_{É¡,q}$ can split FCM.

### Security proof of the introduced SSS utilizing FCM‘

The recommended SSS is based on [39], [40] which is a prevalent signature scheme. When an input message Ïº is provided, it generates  in which  arbitrarily chose its value in a set consist of larger values, D is hash value  and  depends only on , Ïº, and D.

We can derive subsequent standard outcome with the help of direct use of techniques in [40].

Theorem 4.1 *(Forking lemma): Let**be a Turing machine with probabilistic polynomial time, the input of which contains public information only. By R and**, we denote count of relevant queries**may request from the random oracle, and count of relevant queries A may request from the signer, respectively. Suppose in time limit T,**produces a valid (**) signature with a probability of**. If the triple**can be simulated with an indistinguishable probability of distribution without knowing the secret key, then there is another system which has control over the machine which can be obtained from**by replacing the interaction with the signer with a simulation and which produces two valid signatures (*) *and (*) *such that (**) in the predicted time*T'≤120686T/ε*.*

With the help of procedure implemented we acquire two equations in our given technical entry:

**Figure:**
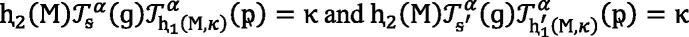

We can get the definite private value with the help of this method

**Figure:**
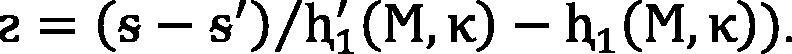

SSS’s security and FCM’s hardness relation get compromised because of ineffectiveness of reductionist technique of Forking lemma. As expected, the secret value  would be obtained when attacker acts in response to the inquiry  by$\kappa T_{t}\left(É¡\right)$, as a substitution for a random number t in $Z_{q}^{*}$ (Note: simulator responds to the -query  (M) by random number t in $Z_{q}^{*}$ as per proof of Theorem 4.1). Hence, oracle replay attack is not required.

As a result, a more dominant reductionist method in depth is supposed to be instigated. A close association among the SSS security and the FCM problem’s hardness can better be shown with the help of following theorem.

Theorem 4.2 *Let*$G_{É¡,q}$*be a (**)-FCM group, then the SSS in the ROM is (**) secure against EUF-CMA, where*

**Figure:**
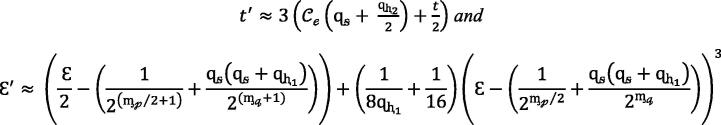

## Here $C_{e}$ refers to the expense of computing a long exponentiation in $G_{É¡,q}$group.

Proof For proving security of SSS, ROM is used. We assume that a EUF-CMA counterfeiter  that () separates the SSS is found. The random oracles , , S can be enquired with a polynomial number of queries by which is a probabilistic polynomial time program arranged with extended open sequence of arbitrary bits.

An algorithm , which receives () as input is needed to be generated for us being a “simulator”. For calculating the FCM i.e.  as a computer programme,  tries to utilize .

Algorithm  simulates one or two SSS runs to counterfeiter A. Hash inquiries  and  are reacted by A, S signature inquiries by Algorithm , and tries to twist A's potential forgeries (Ïº,σ) into an FCM i.e.  solution. By providing () Algorithm  commences the first imitation and an extensive series of arbitrary bits for A. Then, A’s inquiries are responded by as follows:

*Responding**-oracle inquiries*:, To get the compliant answer,  search for the -list (query-response list in which entries contain of tuples $\left(\left({Ïº}_{i},\kappa_{i}\right)D_{i}\right)$ if A subjects a random oracle inquiry $\left({Ïº}_{i},\kappa_{i}\right)$ in which .  replies with $D_{i}$ when tuple $\left(\left({Ïº}_{i},\kappa_{i}\right){,D}_{i}\right)$ is in the -list. Then  homogeneously at random generates $D_{i}$from $Z_{p}^{*}$, responds with it, and improves tuple $\left(\left({Ïº}_{i},\kappa_{i}\right),D_{i}\right)$ to the -list.

*Responding**-oracle inquiries*: In the attempt of achieving the compliant answer,  search for the -list (list of inquiry–response) where entries contain of tuples $(\left({Ïº}_{i}),B_{i},t_{i}\right)$ when  subjects a random oracle inquiry (${Ïº}_{i}$) where . If the -list contains a tuple$(\left({Ïº}_{i}),B_{i},t_{i}\right)$then  reacts with $B_{i}.$

 will search for the -list in the condition of ${(Ïº}_{i})$ is a new inquiry. If the -list contains some tuples$\left(\left({Ïº}_{i},\kappa_{i}\right)D_{i}\right)$,  elects to choose one$\kappa_{i}$, creates $t_{i}$ from $Z_{p}^{*}$ homogeneously at random, evaluates $B_{i}=\kappa_{i}T_{t_{i}}^{\alpha }\left(É¡\right)$ and reacts with $B_{i}$.  adds -list with the tuple$(\left({Ïº}_{i}),B_{i},t_{i}\right)$.  homogeneously creates $t_{i}$ from $Z_{p}^{*}$at random, evaluates $B_{i}=T_{t_{i}}^{\alpha }\left(É¡\right)$ and reacts with $B_{i}$.  adds the -list with the tuple $(\left({Ïº}_{i}),B_{i},0\right)$ in the absence of tuple $\left(\left({Ïº}_{i},\kappa_{i}\right)D_{i}\right)$ in the -list.

*Responding S-oracle inquiries*: For the purpose of obtaining the accurate reply,  search for the S-list (list of query–response) in which entries contain () proviso  subjects an inquiry for signature (${Ïº}_{i}$) in which . When a tuple () occurs in S-list then  retorts with ().

 search for the -list for the first time in the event of ${(Ïº}_{i})$is a new query for signature.  chooses $B_{i}$ if the -list contains a tuple $(\left({Ïº}_{i}),B_{i},t_{i}\right)$ or else  homogeneously generates $t_{i}$ at random from $Z_{p}^{*}$ , calculates $B_{i}=T_{t_{i}}^{\alpha }\left(É¡\right)$, and adds the tuple $(\left({Ïº}_{i}),B_{i},0\right)$to the -list.

Then  homogeneously opts for  from $Z_{p}^{*}$ at random and evaluates .  replies with (), improves the tuple (), to S-list, and improves the tuple ${(Ïº}_{i},D_{i}^{\text{'}},\kappa_{i})$ to -list. If tuple $\left(\left({Ïº}_{i},\kappa_{i}\right)D_{i}\right)$ is in the -list with $D_{i}\neq D_{i}^{\text{'}}$, the simulation will be aborted and restarted (this unfortunate occurrence is at most probability .

We can say that in order to bring entirely distinct outputs contrary to the real attacks oracle based simulations are helpful.

We can presume that a novel authorized message and signature tuple  with probability  are ensued by counterfeiter . When  (M) or  $\left(Ïº,\kappa \right)$ is not inquired by , the probability is , given that both  (M) or  $\left(Ïº,\kappa \right)$ are elected arbitrarily. Thus, the counterfeiter  carries on with the probability  a new signature () such that  and .

The -list consists of two kinds of entries. If , then  implies , and . Considering that the number of -query (Ïº,κ) with  is . Therefore in the first replication the probability of solving the FCM is .

We assume  acquires the signature and message pair () in the first simulation, with  and .

The second simulation with the probability  will be initiated by Algorithm  as long as the same () is supplied. The counterfeiter  is provided with the same random bits series, analogous random responses to hash function and signature queries as those in the first simulation before  requests for  by .

Thus various series of random bits, signatures, and diverse values for random functions tend to be given by . The point to be noted here is that  acts in response with the same value which is at the time of first simulation when the -query $\left({Ïº}_{j}\right)$ is asked after this argument. Here, “Forking lemma” in [40] is applied. We expect that yields signature () this time around such that  and  or the signature  with ${D\text{'}}_{j}\neq D_{j}.$

Here, the “Splitting lemma” [34] is employed to calculate the probability in order to work  as anticipated. Let U be the set of probable random bits series and random function estimates that carry forger  up to the argument where  requests for ; let V be the set of probable random bits series and random function estimates after that. By inference, the probability at which , supplying the series of random bits and random estimates , produces a forgery is  for any ubiquity . Using “Splitting lemma”, a “agreeable” subset occurs Ω∈U such that

(i).
(ii) The probability that A, delivered the arbitrary bits and arbitrary values sequences () in which , produces a copy is at least ε/2.

Expect the sequences of random bit and random function values given up to the argument in first simulation are b. Consequently, the probability that A, delivered(b||v), produces a forgery in second simulation in the condition of any  is .

Forged signature probability  with  and . Forged signature probability  with${D\text{'}}_{j}\neq D_{j}$is .

The probability of  resolving the FCM in the second simulation is thus

**Figure:**
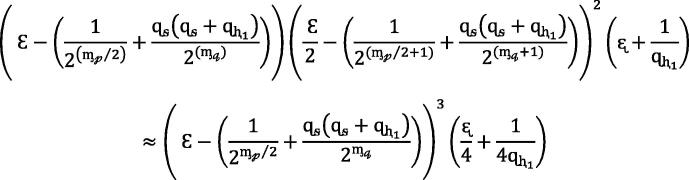

The probabilities can be concluded such that Algorithm  at least solves the FCM with probability (nearly)

**Figure:**
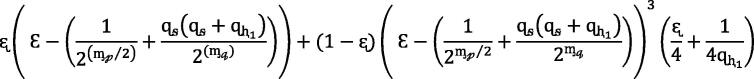

In one simulation the computation stage is . Then final stage in the calculation is

**Figure:**
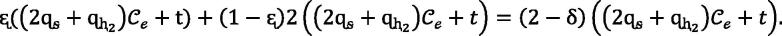

The single -query and one -query are allowed for eachÏº request in the approximation of the probability : akin to the slightly feeble SO-CMA security structure, that is to say, the counterfeiter  demands $\left(Ïº,\kappa \right)$ for both -query and one -query. At the same time Algorithm  reacts with , and . In this case, . Here a robust reductionist evidence of safety is obtained.

Each -query (Ïº,κ) is consequently the -query$\left(Ïº\right)$acting against this affirmative approximation. It leads to find a movable reductionist security evidence as it is observed when  similar to the Schnorr signature scheme. We are eclectically letting  because the set of series of arbitrary bits and arbitrary function values that  supplies to counterfeiter  is arbitrary. Therefore

**Figure:**
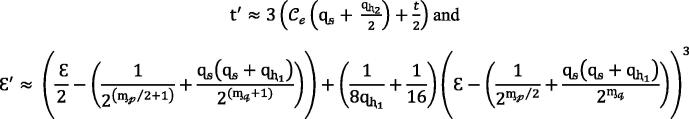

Essentially, this reductionist technique effectively works on the utilization of -query command and -query for the duplicate message insisted by counterfeiter A. Hence, we are led to believe that lying among tight and loose, this reductionist evidence is complete (Goh and Tarecki [36]).

The security of the hash functions: For acquiring a short signature we allow p to be 160 bits. Recovering Ïº and ${Ïº}^{\text{'}}$ messages is unproblematic such that  by birthday attacks consequently the hash value of  is 80 bits. The signature returned by the signer is based on a random number $\kappa^{\text{'}}$instead of κ whenever the attacker insists on a signature on Ïº. Despite the uncertainty of viability in finding other Ïº' message with , it is for certain that finding ${Ïº}^{\text{'}}$ with  is impracticable, as the hash value of  is at least 1024 bits. In the meantime no process will recover  from the multivariate congruence  or find  from . Since the ROM adopts that hash functions are perfect, the probability is

**Figure:**
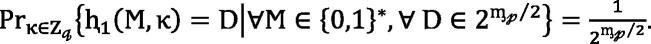

## Performance comparison

In this section, we discussed the performance comparison between the proposed technique and the recent presented technique such as Cui et al.[41], Shen et al. [18], Espositoet al. [42], Mughal et al. [43], Meshram and Obaidat [44] and Zhang et al. [45]. The performance of the proposed work has been discussed based on the storage cost, communication cost, and the computational cost. The performance has been compared based on the cost for signing stage, and the verification stage. Table 2 give information about the notations used for comparative estimations.

**Table 2:** Notations used for comparative estimations.

Sr. No.	Notation	Meaning
1		Execution time for a modular exponentiation in group
2		Execution time for chaotic map operation
3		Execution time for a modular multiplication
4		Execution time for one way hash function
5		Execution time for one bilinear pairing operation
6		Execution time for one modular inverse operation

It has been noted that the signing stage and the verification stage require more computational costs compared to the stage of installation and extraction. Therefore, the comparative study has been done based on the computational cost for signing stage and the verification stage. The state-of-the-art studies discussed in Cui et al. [41], Shen et al. [18], Espositoet al. [42], and Mughal et al. [43], Meshram and Obaidat [44] and Zhang et al. [45] have been compared with the proposed work on performance metrics. The relations between , , , , , and  with respect to  has been established in [25], [26], [27], [46], [47]. The proposed work has used the above mentioned notations and their relations are shown in Table 3.

**Table 3:** Relationship among notations.

Sr. No.	Relationships among notations
1	
2	
3	
4	
5	

Using Table 2, the computational complexity order among the metrics is shown as;

**Figure:**
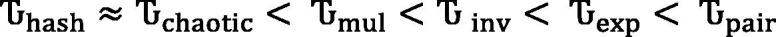

**Fig. 3:**
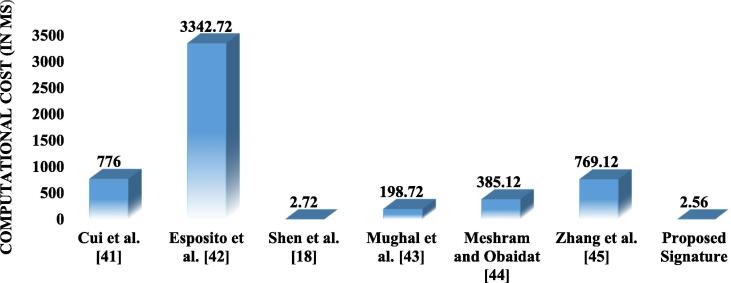
. Comparison based on computational cost for signing stage.

Fig. 3 shows the comparative analysis between the existing schemes and the proposed scheme based on the computational cost for signing stage. The proposed scheme is seen effective as compared to the existing schemes. The proposed scheme requires 2.56 ms for signing stage, shows the effectiveness over the existing schemes.

**Fig. 4:**
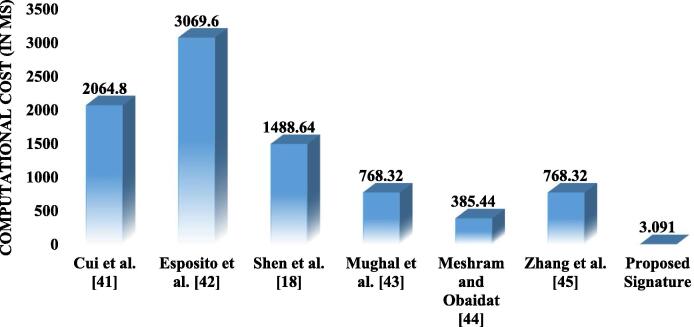
. Comparison based on computational cost for verification stage.

Fig. 4 shows the comparison on the computational cost for verification stage. It shows that the proposed technique is also efficient in verification stage.

Table 4 present the quantitative analysis of the proposed technique and show the comparison based on the total cost including signing stage and the verification stage. It from Table 4 that the total cost has been reduced to 4. 97 ms. Thus, the proposed technique is found to be efficient as compared to the other techniques in the literature.

**Table 4:** Quantitative analysis based on total computational cost including signing stage and verification stage.

Schemes/Stages	Signing stage	Verification stage	Total (ms)
Shen et al. [18]			1489.76
Cui et al. [41]			2840.8
Esposito et al. [42]			5413.12
Mughal et al. [43]			963.84
Meshram and Obaidat [44]			770.56
Zhang et al. [45]			1537.44
Proposed Signature			4.97

## Conclusion

In human-centered IoT, the protection of sensitive data is essential to provide a protection from forgery attacks. Digital signature is the safest option in asymmetric cryptography for ensuring the ownership and validity of the contact parties. This paper uses fractional chaotic maps for secure communication in human-centered IoT to present an effective provably secure short signature technique. This is existentially unforgeable under EUF-CMA at ROM. Results demonstrate the superiority of our strategy, in comparison with competitors, to take fewer overhead based on computing and communication costs alongside resilience studies. The proposed SSS achieves less processing time and less overhead communication in verification and signature operations, in addition to improved resistance to capture attacks. It is therefore very difficult to crack FCM-based SSS compared to DSA which is based on discrete logarithm. In future work, we will develop a new efficient fuzzy signature scheme using fractional chaotic maps for Blockchain using Biometrics under human-centered IoT environments. The limitation of fractional chaotic maps based scheme is only sample selection.

## Compliance with Ethics Requirements

This article does not contain any studies with human or animal data subjects.

## Declaration of Competing Interest

The authors declare that they have no known competing financial interests or personal relationships that could have appeared to influence the work reported in this paper.
